# Letter to the Editor

**DOI:** 10.14309/ctg.0000000000001056

**Published:** 2026-06-03

**Authors:** Mohamad Jamalinia, Amedeo Lonardo

**Affiliations:** 1 Gastroenterohepatology Research Center, Shiraz University of Medical Sciences, Shiraz, Iran;; 2 Independent Researcher, Modena, Italy.

We read with great interest the recent article by Al Ta'ani et al (1), which reported that new-onset heart failure, composite cardiovascular events, and composite cerebrovascular events are higher in lean-metabolic dysfunction-associated steatotic liver disease (MASLD) individuals, compared with their nonlean counterparts. In their sex-stratified analysis in Figure 2, the magnitude of these associations appears greater in men than in women; however, the visually overlapping confidence intervals suggest that this difference is unlikely to be statistically significant (1). Although the sex-specific analysis does not seem to have been adjusted for baseline cardiovascular risk (CVR) factors (1), and therefore it may be premature to draw firm conclusions, these findings may still provide useful insights into potential sex differences in MASLD-related cardiovascular risk.

In a meta-analysis of 36 cohort studies with aggregate data on ∼18.5 million individuals, our group (2) showed that, over a median follow-up of 6.9 years, MASLD was associated with a higher risk of fatal and/or nonfatal cardiovascular events (e.g., myocardial infarction, angina pectoris, heart failure, ischemic or hemorrhagic strokes, or coronary revascularization procedures) in women than in men. Greater MASLD severity further increased this risk, which was more pronounced in women (2). The results were unchanged in sensitivity analyses (2).

Direct comparison of the significantly higher MASLD-related CVR observed in women in our meta-analysis (2) with the nonsignificantly higher lean-MASLD–related CVR in the opposite direction (i.e., in men) in the multicenter study by Al Ta'ani (1) raises intriguing considerations. It can indeed be speculated that it is the adipose tissue dysfunction that may partly underlie the higher MASLD-related CVR in women than in men. Obesity-driven adipose tissue dysfunction contributes to chronic inflammation and cardiovascular pathogenesis through a shift toward proinflammatory and away from anti-inflammatory adipokines (3). However, the pathobiological significance of these mechanisms is probably underappreciated in the context of sex-specific MASLD-related CVR. If confirmed by prospective studies, these findings could inform targeted sex-specific preventive and therapeutic strategies. Notably, several pharmacological approaches—including incretin mimetics (semaglutide and tirzepatide), PPAR-gamma agonists (lanifibranor), and FGF21 analogs (efruxifermin and pegozafermin)—act on adipose tissue to treat MASLD and reduce CVR (4).

Finally, Al Ta'ani et al (1) were unable to evaluate the impact of disease severity or fibrosis progression on cardiovascular outcomes. In this context, a previous meta-analysis showed that even early stages of liver fibrosis (≥F1) are associated with subclinical atherosclerosis in patients with MASLD, with stronger associations observed at higher fibrosis stages (5). Assuming that similar relationships extend to clinical cardiovascular outcomes (2), further studies are warranted to clarify the interplay between adiposity and liver fibrosis (5) in shaping sex-specific CVR in MASLD and its assessment.

## CONFLICTS OF INTEREST

**Guarantor of the article:** Mohamad Jamalinia, MD, MPH.

**Specific author contributions:** M.J.: accepting full responsibility for the integrity of the work and the decision to publish. A.L.: drafted the first version of the manuscript. A.L. and M.J.: reviewed and revised the manuscript and provided critical input.

**Financial support:** None to report.

**Potential competing interests:** M.J. and A.L. have nothing to declare.
